# ERK and phosphoinositide 3-kinase temporally coordinate different modes of actin-based motility during embryonic wound healing

**DOI:** 10.1242/jcs.133421

**Published:** 2013-11-01

**Authors:** Jingjing Li, Siwei Zhang, Ximena Soto, Sarah Woolner, Enrique Amaya

**Affiliations:** 1 Faculty of Life Sciences, University of Manchester, Oxford Road, Manchester M13 9PT, UK; 2 The Healing Foundation Centre, Michael Smith Building, University of Manchester, Oxford Road, Manchester M13 9PT, UK

**Keywords:** ERK, PI3K, Wound healing, Rho GTPases, *Xenopus*

## Abstract

Embryonic wound healing provides a perfect example of efficient recovery of tissue integrity and homeostasis, which is vital for survival. Tissue movement in embryonic wound healing requires two functionally distinct actin structures: a contractile actomyosin cable and actin protrusions at the leading edge. Here, we report that the discrete formation and function of these two structures is achieved by the temporal segregation of two intracellular upstream signals and distinct downstream targets. The sequential activation of ERK and phosphoinositide 3-kinase (PI3K) signalling divides *Xenopus* embryonic wound healing into two phases. In the first phase, activated ERK suppresses PI3K activity, and is responsible for the activation of Rho and myosin-2, which drives actomyosin cable formation and constriction. The second phase is dominated by restored PI3K signalling, which enhances Rac and Cdc42 activity, leading to the formation of actin protrusions that drive migration and zippering. These findings reveal a new mechanism for coordinating different modes of actin-based motility in a complex tissue setting, namely embryonic wound healing.

## Introduction

Wound healing is a complex process in which diverse cell and tissue lineages must work together to restore skin integrity and homeostasis following injury (Martin, 1997). Embryos have remarkable capabilities to repair wounds rapidly and perfectly and, unlike adult wounds, embryonic wounds heal without scar formation. Therefore, the study of wound repair in embryos has become an important system for investigating the healing process and developing therapeutic applications to improve adult wound healing. There are several differences between wound healing in embryonic and adult tissues, which could contribute to the improved healing capacity of embryos. These include differences in the inflammatory response, cell proliferation and distinct mechanisms of re-epithelialisation (Nodder and Martin, 1997).

Re-epithelialisation in adult wounds is characterised primarily by the crawling of cells that surround the wound (leading-edge cells) over the exposed wound substratum. This movement is propelled by actin-based sheet-like membrane projections called lamellipodia (reviewed by Martin, 1997). By contrast, embryos heal wounds primarily by assembling a contractile actin cable at the wound margin, which functions to draw the epithelium and connective tissue forward together in a ‘purse-string-like’ fashion (Martin and Lewis, 1992). Along with the cable, embryonic leading edge cells also form dynamic actin protrusions, finger-like filopodia and sheet-like lamellipodia, which work in concert with the cable to close the wound (Davidson et al., 2002; Wood et al., 2002). The purse string structure is not entirely restricted to embryonic wound healing, because actomyosin cables have been observed in wounds of adult gut and cornea epithelia (Bement et al., 1993; Danjo and Gipson, 1998). Analysis of the actin cytoskeleton structures that are formed during tissue repair has revealed similarities with some morphogenetic processes that occur during normal embryonic development (Martin and Parkhurst, 2004). For example, during *Drosophila* dorsal closure, in which a large hole in the embryonic epithelium is closed, leading edge cells first accumulate an actomyosin cable, which draws the opposing epithelial sheets together. Closure is then completed by a process called ‘zippering’, in which lamellipodia and filopodia help to fuse the epithelium shut (Young et al., 1993; Jacinto et al., 2000; Kiehart et al., 2000; Jacinto et al., 2002). These same actin structures are also adopted by *C. elegans* embryos during ventral enclosure (Williams-Masson et al., 1997; Chin-Sang and Chisholm, 2000; Simske and Hardin, 2001). The similarities between morphogenetic events and tissue repair, especially embryonic tissue repair, mean that wound healing can also be considered a re-activation of morphogenetic processes prevalent during gastrulation.

An essential question in both embryonic wound closure and similar morphogenetic processes is what signals initiate and coordinate the formation of the distinct cytoskeletal machineries that drive the tissue movements. It has been shown that injury triggers an activation of Rho and Cdc42 (Clark et al., 2009), and *in vitro* and *in vivo* studies have suggested that the small GTPases Rac, Cdc42 and Rho play crucial but distinct roles in regulating actin dynamics. Rho regulates the formation of stress fibres and contractile cables (Ridley and Hall, 1992; Wood et al., 2002), Cdc42 promotes filopodia formation (Nobes and Hall, 1995; Wood et al., 2002) and Rac drives membrane ruffling, lamellipodia formation and actin accumulation at the leading edge (Ridley et al., 1992; Woolner et al., 2005). However, whereas we know much about these direct regulators of the actin cytoskeleton, much less is known about the upstream signals involved in wound closure and, moreover, how they are coordinated in time and space to regulate functionally distinct downstream targets.

Here, we use the *Xenopus* embryo as our *in vivo* embryonic wound-healing model, and discover two distinct phases of wound closure that are controlled by sequential activation of extracellular signal-regulated kinase (ERK) and phosphoinositide 3-kinase (PI3K) signalling. ERK activity initiates the first phase, leading to a peak of Rho activity and subsequent actomyosin purse-string assembly and contraction. PI3K activity is suppressed by ERK signalling in this phase, and restored upon ERK attenuation. Restored PI3K signalling predominates the second phase, specifically elevating Rac and Cdc42 activity and promoting filopodia formation at the wound edge for migration and zippering. The exquisite coordination of these two intracellular upstream signalling pathways determines a temporal segregation of the functionally distinct Rho GTPases and their cytoskeleton targets, revealing a novel mechanism for orchestrating different tissue movements in both wound healing and morphogenesis.

## Results

### Embryonic wound closure comprises two distinct temporal phases

**Fig. 1.**
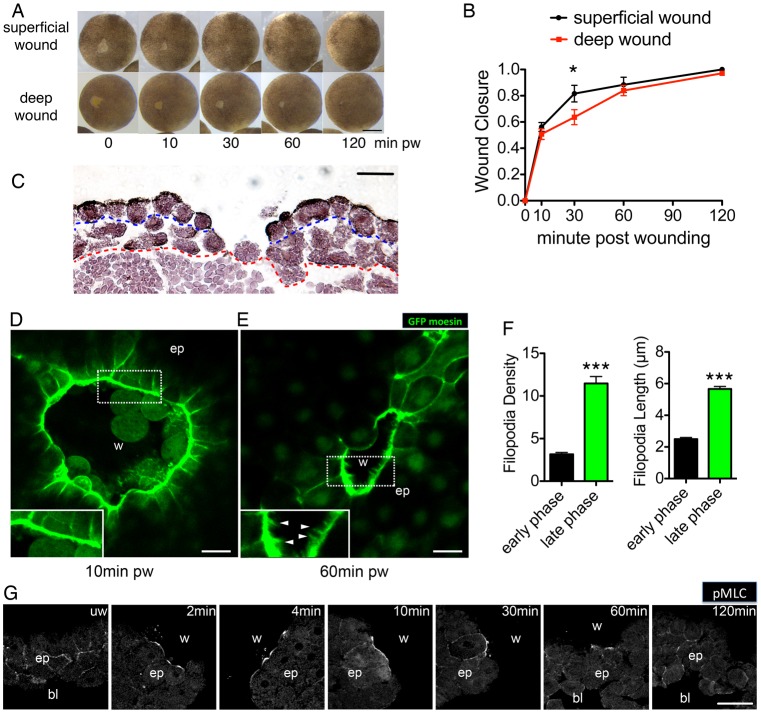
**Embryonic wound healing comprises distinct temporal phases.** (A) Time-lapse series of superficial and deep wound closure in blastula stage embryos. pw, post wounding. (B) Quantification of wound closure speed in superficial and deep wounds. Data are means ± s.d. of three independent experiments, *n* = 5 embryos for each experiment. Two-way ANOVA was performed to confirm significance. *P<0.05. (C) H&E staining of a deep wound at 1 hour after wounding. Blue dashed line represents boundary between superficial (epithelial) layer and deep (mesenchymal) layer on the animal cap. Red dashed line indicates border between the animal cap cells and vegetal cells of the embryo. Note that the deep layer has healed close by this time point, whereas the superficial layer has not yet healed. (D) Circumferential actin cable at the wound edge 10 minutes post wounding in GFP-moesin-injected embryo. Inset shows thick actin cable with few or no filopodial protrusions. (E) Filopodia formation at the wound edge 60 minutes after wounding in GFP-injected embryo. Inset shows proximal wound fronts with filopodia. Arrows indicate filopodia extended from opposing wound edges. w, wound area; ep, epithelium. (F) Quantification of the density (number of filopodia per 100 µm) and length of filopodia at the wound edge in early and late phases (means ± s.e.m.; ***P<0.001. (G) Time course of immunofluorescence staining for Ser19 phosphorylation on myosin-2 light chain in transected wounds. w, wound; bl, blastocoel; ep, epithelium. Scale bars: 500 µm (A), 50 µm (C,G), 10 µm (D,E).

Owing to their large size and external development, *Xenopus* embryos have proved to be a powerful vertebrate system in wound-healing research (Stanisstreet, 1982; Clark et al., 2009; Fuchigami et al., 2011). To understand the regulation of cell dynamics in embryonic wound healing, we started by characterising tissue movement of late blastula stage wound closure, in which two types of excisional wounds could be compared. At this stage, the animal cap consists of two to three layers of prospective ectoderm cells: a superficial, epithelial cell layer and a deep, mesenchymal cell layer (Davidson et al., 2002). First we generated a superficial wound in the animal cap, by removing a small region of the superficial cell layer using forceps. Wound closure of these superficial wounds began with a fast early phase, which lasted 30 minutes. During the early phase it took only 10 minutes for the wound to close 50% and 30 minutes to close up to 80%. Following the early phase, a slow second phase began, in which the wound took nearly 90 minutes to heal the remaining 20% of the wound area (Fig. 1A, top panel; Fig. 1B). To act as a comparison to our superficial wounds, we also generated deep wounds, in which the deeper layer of supporting mesenchymal cells was removed together with the superficial layer. We observed that in the first 10 minutes of closure, the wound closure curve of deep wounds was similar to that of the superficial wounds (Fig. 1A, bottom panel; Fig. 1B). However, between 10 and 60 minutes following wounding, the progression of deep wound closure was delayed, due to a ‘transition’ phase, which coincided with the repair of the deep layer of mesenchymal cells (Fig. 1C). Once the mesenchymal layer healed, wound closure then proceeded at the same rate as for superficial wounds (Fig. 1B). The comparison between superficial and deep wound healing suggested the involvement of distinct mechanisms during the different phases of wound healing. In the early phase, it appeared that an intact deep layer is not essential for powering normal healing, because both superficial and deep wounds closed at the same rate. By contrast, the support of an intact mesenchymal layer seemed indispensable in the slow phase, because a delayed ‘transition’ phase in deep wound closure coincided with healing of the deep layer, before initiation of the slow phase.

### The early phase of embryonic wound healing is characterised by actomyosin-driven contraction, whereas the late phase is characterised by filopodial-based migration and zippering

During embryonic wound healing, leading edge cells re-organise their actin cytoskeleton to form two structures that facilitate tissue movement: a contractile actin cable and lamellipodial/filopodial protrusions. The contractile actin cable comprises F-actin and myosin-2, and functions to contract the wound edge in a purse-string manner (Martin and Lewis, 1992); whereas lamellipodial and filopodial protrusions assist migration and zippering of opposing cell sheets (Davidson et al., 2002; Wood et al., 2002). To examine actin dynamics during the different phases of *Xenopus* wound closure, we injected albino *Xenopus laevis* embryos with mRNA encoding GFP-tagged moesin (F-actin binding domain) and live imaged actin dynamics during closure of superficial wounds. We found that a circumferential F-actin ring emerged at the wound edge within minutes of wounding (Fig. 1D; supplementary material Movies 1,2), coinciding with the early phase of embryonic wound healing. Only a few filopodia were formed at the leading edge during this period. Once the wound reached the late phase of closure (1 hour post wounding), we observed greater numbers of filopodia at the wound leading edge (Fig. 1E,F). These filopodia were also longer than those during the early phase (Fig. 1F) and appeared to be zippering the two opposing sheets together (supplementary material Movies 3,4).

We next analysed the localisation and activation of non-muscle myosin-2, the other component of the actomyosin purse string. Immunostaining of phosphorylated Ser19 of the regulatory light chain of myosin-2 (pMLC) (Benink and Bement, 2005) revealed that active myosin-2 accumulated at the wound edge from 2 minutes post wounding, reaching a peak at 10 minutes, which was sustained for more than 1 hour (Fig. 1G). This timing of active myosin-2 staining correlated well with the formation of actin cable and the early phase of wound closure. Taken together, these findings indicate that the formation of an actin cable and actomyosin contraction provide the major driving force during the early phase of wound closure. By contrast, the late phase is characterized by an increase in filopodial protrusions as the wound edges zipper together.

### ERK and PI3K signalling are sequentially activated in distinct phases during embryonic wound healing

**Fig. 2.**
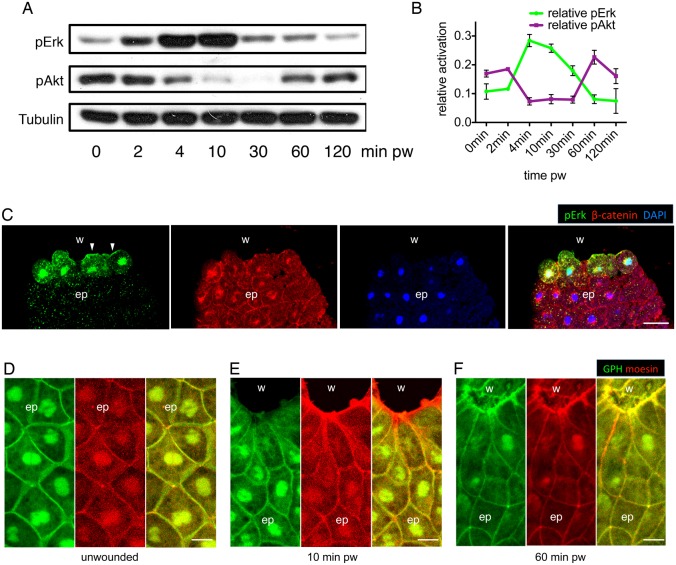
**ERK and PI3K signalling are sequentially activated during embryonic wound healing.** (A) Western blot analysis shows the sequential activation of pERK and pAkt in deep wounds, with α-tubulin as loading control. (B) Quantification of pERK and pAkt activation during embryonic wound healing. Data are means ± s.e.m. from three independent experiments and represent pERK and pAkt signal intensities normalized to α-tubulin controls. (C) Immunofluorescence staining of pERK, β-catenin (plasma membrane) and DAPI (nucleus) on a transected embryonic wound 10 minutes after wounding. w, wound; bl, blastocoel; ep, epithelium. (D–F) PIP3 localisation in unwounded epithelium (D), early phase wound (E) and late phase wound (F). Green, GFP-Grp1; red, mCherry-moesin; w, wound; ep, epithelium; pw, post wounding. Scale bars: 50 µm (C), 20 µm (D–F).

To investigate how the temporal phases of wound closure might be controlled, we began to analyse signalling pathways that are known to be activated following injury. Previous work in *Drosophila* has shown that ERK/MAPK can be activated by two upstream signalling pathways post wounding. One of these pathways involves Stitcher, a Ret family receptor tyrosine kinase (RTK) that activates ERK signalling and the transcription factor Grainy head, and is required for actin cable formation (Wang et al., 2009; Kim and McGinnis, 2011). The other is the platelet-derived growth factor/vascular-endothelial growth factor (PDGF/VEGF), through its receptor, Pvr, which leads to the activation of ERK in embryos (Cho et al., 2002) and is required for larval wound edge actin assembly and wound repair (Wu et al., 2009). In *Xenopus* embryos, activation of the ERK/MAPK signalling pathway is also one of the early wound responses (LaBonne et al., 1995; Christen and Slack, 1999). To explore the timeframe of ERK activation during embryonic wound closure, we assessed the phosphorylation of ERK on Thr202/Tyr204 (pERK). In both superficial and deep wounds, ERK activation was detectable from 2 minutes after wounding by western blot analysis (Fig. 2A; supplementary material Fig. S1A). The phosphorylation remained high for up to 30 minutes and decreased by 1 hour (Fig. 2A,B; supplementary material Fig. S1A,B). Immunostaining showed a similar timescale of ERK activation (supplementary material Fig. S1C), and in particular, immunostaining revealed an accumulation of pERK at the leading edge plasma membrane and nuclei of wound edge cells (Fig. 2C). Activation of ERK therefore appeared to overlap temporally with the early phase and spatially with the contractile actin cable in wound edge cells.

We next examined PI3K signalling, another essential pathway in cell migration. Intriguingly, we found that the level of PI3K signalling, assessed by detection of phosphorylated Akt (Ser473) (pAkt) using western blot analysis, declined coincidentally with the rise of ERK signalling in both superficial and deep wounds (Fig. 2A; supplementary material Fig. S1A). PI3K signalling was restored to original levels when ERK activation ceased, 1 hour post wounding (Fig. 2A,B; supplementary material Fig. S1A,B), overlapping with the late phase of embryonic wound healing. We also assessed PI3K activity in the wound area using a GFP-tagged PH domain of Grp1 (GPH), which binds to the product of PI3K, phosphatidylinositol 3,4,5-trisphosphate (PIP3), on the plasma membrane (Pickering et al., 2013). Injection of mRNAs encoding a dominant-negative version of PI3K (Δp85) and a constitutively active version of PI3K (p110 CAAX) (Carballada et al., 2001) served as controls to confirm the efficiency of the GPH probe (supplementary material Fig. S1D). We discovered that during the early phase of wound closure, there was a remarkable decrease in GPH accumulation at the leading edge (Fig. 2D,E). However, the PI3K activity, as measured by GPH accumulation, was restored to a high level at the leading edge in the late phase (Fig. 2F), indicating a temporal as well as spatial inverse activation with ERK signalling.

### ERK and PI3K signalling regulate different phases of embryonic wound closure

**Fig. 3.**
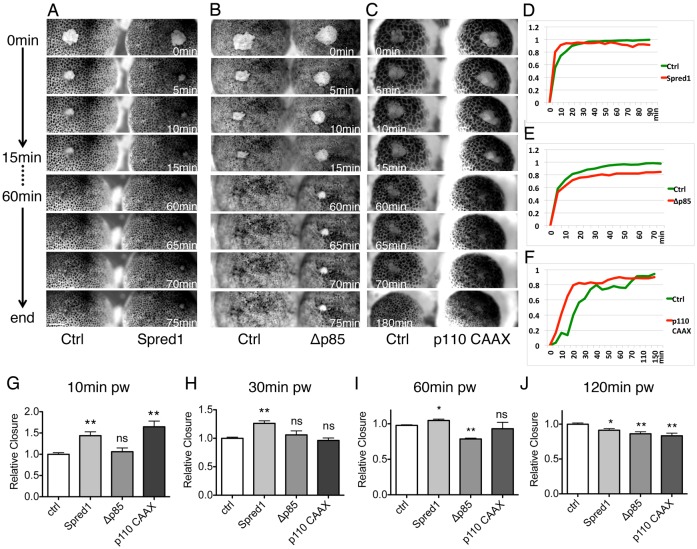
**ERK and PI3K signalling regulate distinct phases of wound healing.** (A–C) Time-lapse series of wound healing in control and in embryos injected with *spred1* (A), *Δp85* (B) and *p110 caax* (C). Each image shows time post wounding. Scale bar: 200 µm. (D–F) Healing curves of results in A–C. (G–J) Quantification of relative wound closure 10 minutes (G), 30 minutes (H), 60 minutes (I) and 120 minutes (J) post wounding. Mean wound closure percentage of control embryos was normalized to 1; other groups show closure relative to control. Results are shown as means ± s.e.m.; *n* = 3. Non-parametric Mann–Whitney test was used to test for significance between control and other groups. *P<0.05; **P<0.01; ***P<0.001; ns, not significant.

Our finding that ERK and PI3K signalling are activated sequentially, overlapping with the early and late phases respectively, suggested that the two pathways might act functionally in the two phases of embryonic wound healing. To test this hypothesis, we first disrupted ERK signalling by injecting *spred1* mRNA (Sivak et al., 2005), thus inhibiting signalling transduced from Ras to Raf (supplementary material Fig. S2A) (Wakioka et al., 2001). Unexpectedly, embryos injected with *spred1* mRNA showed remarkable acceleration in the early phase of healing compared with the control, by closing 80% of the wound area in 10 minutes (Fig. 3A, top panel; Fig. 3D,G; supplementary material Fig. S2C, Movie 5). However, from 1 hour onward, they lost the advantage of wound closure and finished with a gaping-open wound (Fig. 3A bottom panel; Fig. 3D,J; supplementary material Fig. S2C′). To confirm this phenotype, we used a chemical inhibitor of MEK1, PD184352 (PD), to inhibit ERK signalling. Similar to *spred1* overexpression, PD treatment also accelerated the early phase of wound closure and the effect was reversed at the last phase of closure (supplementary material Fig. S2F–I).

To modulate PI3K signalling, we used three tools: the dominant-negative PI3K (Δp85) construct and a chemical inhibitor of PI3K (LY294002) to disrupt PI3K activity; and the constitutively active PI3K (p110 CAAX) construct to hyper-activate PI3K signalling (supplementary material Fig. S2B). When PI3K signalling was inhibited by injecting *Δp85* mRNA, we observed no significant difference in the early phase of healing (Fig. 3B, top panel; Fig. 3E,G,H; supplementary material Fig. S2D). However, as wound closure progressed, *Δp85*-injected embryos revealed a remarkable delay in the late phase of healing, with a complete failure of wound zippering (Fig. 3B, bottom panel; Fig. 3E,I,J; supplementary material Fig. S2D′, Movie 6). Equivalent results were obtained following treatment of healing embryos with LY294002 (LY), confirming that inhibition of PI3K signalling exhibited a normal early phase of wound closure (supplementary material Fig. S2F,G), but failed to conclude the late phase of closure (supplementary material Fig. S2H,I). By contrast, when we artificially elevated PI3K activity by injecting *p110 CAAX* mRNA, an accelerated early phase was observed (Fig. 3C, top panel; Fig. 3F,G; supplementary material Fig. S2E, Movie 7). However embryos injected with *p110 CAAX* mRNA appeared to have high mortality upon gastrulation, which could account for the defect seen at the very end of wound closure (Fig. 3J).

### ERK signalling suppresses PI3K signalling in the early phase

The result that inhibition of ERK could accelerate wound healing in the early phase contradicts previous reports suggesting a positive role for ERK signalling during wound closure (Matsubayashi et al., 2004). To explain this unusual finding, we noticed an unexpected similarity between the effects of ERK inhibition and PI3K hyper-activation in the early phase, and speculated that ERK signalling might normally restrict PI3K signalling in the early phase, thus limiting the PI3K-mediated advancement of the leading edge cells during this phase.

**Fig. 4.**
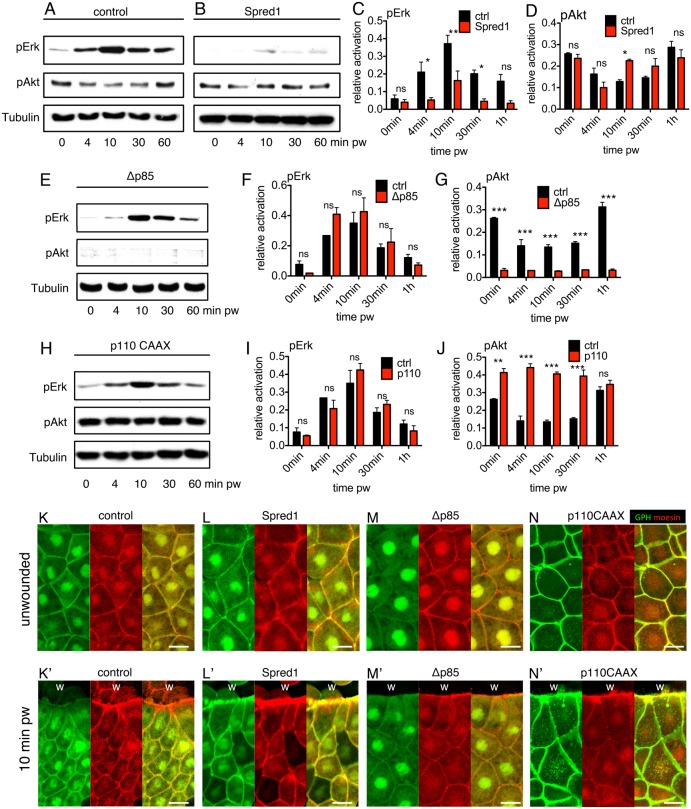
**Disruption of ERK signalling results in a quicker restoration of PI3K signalling.** (A–D) Western blots and quantification of pERK and pAkt in *spred1* and control injected embryos post wounding. (E–G) Western blot and quantification of pERK and pAkt in *Δp85* and control injected embryos post wounding. (H–J) Western blot and quantification of pERK and pAkt in *p110-caax-* and control-injected embryos post wounding. For all western blot quantifications, α-tubulin was the loading control. For the quantification graphs, control bars (black) are pERK or pAkt levels in control embryo at different time points. Red bars are pERK or pAkt levels of experimental groups at different time points. Values are means ± s.e.m.; *n* = 3. pw, post wounding. *P<0.05; **P<0.01; ***P<0.001; ns, not significant. (K–M) PIP3 localisation in control (K,K′), Spred1 (L,L′), Δp85 (M,M′) and p110 CAAX (N,N′) embryo. K–N show unwounded epithelium, K′–N′ show the leading edges. w, wound; green, GFP-Grp1; red, mCherry-moesin. Scale bars: 20 µm.

Sequential activation of ERK and PI3K pathways has been previously described during growth factor signalling (Foschi et al., 1997) and was later shown to be mediated by an ERK-dependent negative regulation of PI3K activity (Hayashi et al., 2008). However, this phenomenon has never been described during wound repair, so we set out to investigate whether a similar relationship existed in our wound-closure system. Using western blot analysis, we found that *spred1* overexpression did not alter the immediate decrease in PI3K signalling 4 minutes post wounding, but it did result in a precocious, rapid restoration of pAkt after 10 minutes, whereas controls only restored pAkt after 1 hour (Fig. 4A–D). To rule out the possibility that Spred1 itself acts as a direct negative regulator of PI3K signalling in addition to its function as a Ras and ERK inhibitor, we used the chemical inhibitor of ERK signalling, PD. Similar to injection of *spred1* mRNA, PD treatment also caused a quick restoration of PI3K signalling at 10 minutes post wounding (supplementary material Fig. S3A–D), confirming the existence of ERK-dependent negative regulation of PI3K signalling in the sequential activation of ERK and PI3K. As previous studies also suggested an inverse negative regulation from PI3K to ERK signalling (Hayashi et al., 2008), we modulated the level of PI3K signalling with Δp85, LY and p110 CAAX. Although all three tools altered PI3K activity as expected (Fig. 4E,G,H,J; supplementary material Fig. S3E,G), the modulated PI3K signalling did not affect the dynamics of ERK activation (Fig. 4E,F,H,I; supplementary material Fig. S3E,F), suggesting that the crosstalk between ERK and PI3K signalling during wound healing is one-directional. Parallel experiments were performed with the GPH probe and showed that whereas control embryos exhibited reduced PI3K activity at the leading edge in the early phase (Fig. 4K,K′), inhibiting ERK signalling by overexpressing *spred1* led to high PI3K activity at the wound front (Fig. 4L,L′). Similar activation of PI3K at the leading edge could be observed in embryos injected with *p110 CAAX* (Fig. 4N,N′), whereas Δp85 strongly attenuated PI3K activity (Fig. 4M,M′). Therefore, ERK activation in the early phase of wound closure suppresses PI3K signalling, and the shut down of ERK signalling in the late phase permits a recovery of PI3K signalling.

To assess whether the acceleration in the speed of wound closure when ERK signalling was artificially attenuated was dependent on PI3K, we first combined a medium dose of Δp85 or LY (supplementary material Fig. S4E,F) with Spred1 or PD, which achieved a moderate double inhibition of PI3K and ERK signalling, and discovered that it eliminated the accelerated effect by ERK inhibition on wound closure (supplementary material Fig. S4A,B), ending with a delayed late phase (supplementary material Fig. S4C,D). Furthermore, a combination of a high dose of Δp85 or LY (supplementary material Fig. S4E,F) and Spred1 further slowed down the early phase (supplementary material Fig. S4G–J), indicating that in the absence of activated ERK, precocious PI3K activity acts as the driving force for wound closure.

### ERK signalling modulates actomyosin contraction and PI3K regulates filopodia extension at the leading edge

The above wound closure results suggested that PI3K signalling normally acts mainly in the late phase of embryonic wound closure, playing a positive role; whereas the function of ERK signalling is more complicated: it is required to avoid a gaping-open wound, but it also strongly restricts PI3K-mediated closure in the early phase. Therefore, we wondered which, if any, of the actin-based structures were regulated by ERK and PI3K signalling during the different phases of embryonic wound closure.

**Fig. 5.**
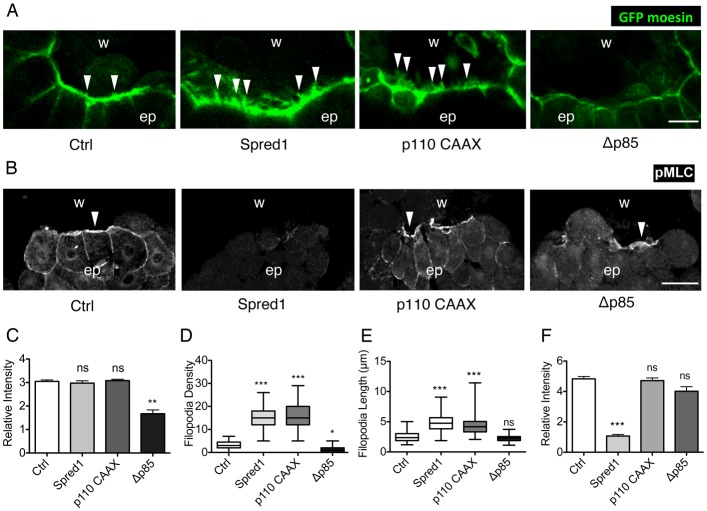
**ERK regulates myosin-2 phosphorylation and PI3K promotes actin accumulation and filopodia formation at the wound edge.** (A) Confocal images of actin cable and filopodia at the wound edge in GFP-moesin-injected embryos in combination with control, *spred1*, *p110caax* and *Δp85* mRNA injections, 10 minutes post wounding. Arrowheads indicate filopodia. Scale bar: 20 µm. (B) Immunofluorescent staining of pMLC on transected wounds in control uninjected embryos and embryos injected with *spred1*, *p110caax* and *Δp85*. Embryos were fixed 10 minutes post wounding. w, wound area; ep, epithelium. Arrowheads indicate pMLC at the wound edge. Scale bar: 50 µm. (C) Quantification of relative F-actin fluorescence intensity at the wound edge. *n* = 20 cells from 10 embryos from three batches were measured. (D) Quantification of filopodia number per 100 µm in wounds. *n*_ctrl_ = 65, *n*_Δp85_ = 44, *n*_p110_ = 75, *n*_spred1_ = 51 (×100 µm of wound edge) of at least 20 embryos from three batches were analysed. Data are plotted as box and whisker plots. (E) Quantification of filopodial length in wounds. *n*_ctrl_ = 63, *n*_Δp85_ = 85, *n*_p110_ = 122, *n*_spred1_ = 105 (filopodia) on at least 20 embryos from three batches were analysed. Data are plotted as box and whisker plots. (F) Quantification of relative pMLC fluorescence intensity at the wound edge. *n* = 20 wound edge cells from at least three batches of embryos were analysed. All results are shown as means ± s.e.m. and Kruskal–Wallis one-way ANOVA test was used to compare all groups to control. *P<0.05, **P<0.01, ***P<0.001; ns, not significant.

Analysis of actin dynamics showed that control embryos (supplementary material Movie 8), *spred1*-mRNA-injected embryos (supplementary material Movie 9) and *p110-CAAX*-mRNA-injected embryos (supplementary material Movie 10) assembled an actin cable at the wound edge 10 minutes after wounding (Fig. 5A). By contrast, embryos injected with *Δp85* mRNA showed impaired F-actin accumulation (Fig. 5A; supplementary material Movie 11) over the same time scale. This finding was confirmed by quantification of the F-actin fluorescence intensity (FI) at the leading edge, which showed a 50% decrease in PI3K-inhibited embryos (Fig. 5C).

The formation of filopodial protrusions was also significantly altered upon modulation of ERK and PI3K signalling. Control embryos formed only a limited number of filopodia at the wound margin in the early phase of wound closure. However, when ERK signalling was inhibited, significantly more and longer filopodia were formed at the leading edge (Fig. 5A,D,E). Disruption of PI3K activity reduced the number (Fig. 5D), but not the length of filopodia (Fig. 5E). When we artificially hyper-activated PI3K, filopodia number dramatically increased (Fig. 5D), together with a significant increase in filopodial length, from 2.5 µm to 5 µm (Fig. 5E).

We next examined the phosphorylation of myosin-2 (pMLC), the driving force of actomyosin contraction. Control embryos revealed strong pMLC accumulation at the wound margin (Fig. 5B), whereas inhibiting ERK activation, by either injection of *spred1* mRNA or PD treatment, eliminated pMLC accumulation to the leading edge (Fig. 5B,F; supplementary material Fig. S5). By contrast, disruption of PI3K signalling upon *Δp85* mRNA injection or LY treatment, or enhancement of PI3K signalling by injecting *p110 CAAX* mRNA did not significantly change the level of pMLC accumulation (Fig. 5B,F; supplementary material Fig. S3).

In summary, we found that ERK is required for activation of myosin-2 and therefore actomyosin contraction; and PI3K is involved in filopodial protrusion. Loss of ERK leads to an overproduction of filopodia.

### ERK and PI3K signalling regulate cytoskeleton organisation through small Rho GTPases

Having established the distinct effects of ERK and PI3K signalling on cytoskeletal dynamics at the wound edge, we next sought to identify the downstream effectors that link the upstream signalling events to the downstream cytoskeletal components. *In vitro* experiments have shown that both ERK and PI3K signalling are able to modulate nuclear transcription factors in wound edge cells (Fitsialos et al., 2007). When we analysed pERK in embryonic wounds by immunostaining, we found that it colocalised with the nuclei in wound edge cells (supplementary material Fig. S6A), suggesting that transcriptional targets could be regulated by ERK signalling during embryonic wound closure. Hence, we first examined whether transcription was required for embryonic wound closure. We injected embryos with α-amanitin at a concentration known to inhibit *Xenopus* polymerase II transcription (Biddle et al., 2009). Real-time PCR on *siamois* and *xnr3*, two genes transcribed at the midblastula transition, showed that α-amanitin effectively inhibited transcription in embryos (supplementary material Fig. S6B). However, we found that embryos injected with α-amanitin did not show a significant defect in wound closure (supplementary material Fig. S6C,D), indicating that transcription was not required for embryonic wound healing. Therefore, ERK and PI3K signalling are unlikely to regulate embryonic wound closure at the transcriptional level. This finding fits with the speed of cytoskeletal reorganisation seen during embryonic wound healing, which seems too fast to be controlled at the transcriptional level (Clark et al., 2009).

The Rho family of small GTPases are key regulators of actin dynamics during tissue repair, and are therefore good candidates for linking ERK and PI3K signalling to actin assembly. We therefore investigated whether ERK and PI3K signalling could target directly the Rho family members Rac, Rho and Cdc42. We developed an active Rho GTPase GST pull-down assay applicable to our *in vivo* embryonic wound-healing system, to detect the levels of active Rac, Rho and Cdc42 (supplementary material Fig. S7) (Soto et al., 2013). The binding domain of PAK1 (aa 70–117) was used for pulling down active Rac and Cdc42 (Zhang et al., 1998), and the binding domain of Rhotekin for active Rho (Benink and Bement, 2005).

**Fig. 6.**
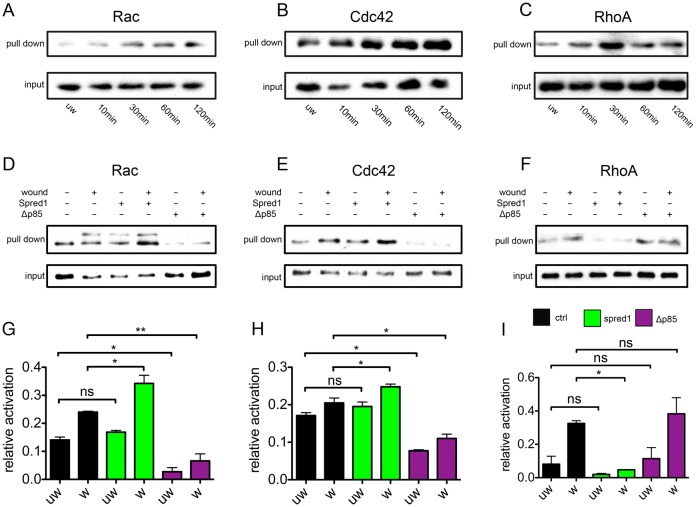
**Small Rho GTPases Rac and Cdc42 are regulated by PI3K signalling, whereas Rho is regulated by ERK signalling during wound healing.** (A–C) Active Rac (A), Cdc42 (B) and Rho (C) GST pull-downs in control embryos during wound healing. 5% embryo lysate was taken as input. (D–F) Active Rac (D), Cdc42 (E) and Rho (F) GST pull-downs in control and in embryos injected with *spred1* and *Δp85* during wound healing. 5% embryo lysate was taken as input. (G–I) Quantification of D–F. Spred1 significantly increases Rac (G) and Cdc42 (H) activation, and reduces Rho activity (I) post wounding. Δp85 reduces Rac (G) and Cdc42 (H) activity before and post wounding, but does not affect Rho activity (I). All results are shown as means ± s.e.m.; *n* = 3. w, wounded; uw, unwounded. *P<0.05, **P<0.01; ns, not significant.

We started by analysing the activation state of Rac, Cdc42 and Rho during the entire wound-healing process. Both Rac and Cdc42 were moderately activated immediately post injury (Fig. 6A,B, 10 minutes post wounding). Moreover, the activation was gradually enhanced as wound healing progressed, reaching a peak in the final stage (Fig. 6A,B, 60 minutes and 120 minutes post wounding). Similar to Rac and Cdc42 activation, activation of Rho occurred immediately post wounding (Fig. 6C, 10 minutes post wounding). However, it reached its peak at 30 minutes, and started to decrease, although still maintained at a higher level than unwounded embryos (Fig. 6C, 60 minutes and 120 minutes post wounding). Intriguingly, activation of Rho temporally correlated with ERK phosphorylation in the early contraction phase of wound closure, whereas the peak of Rac and Cdc42 activation overlapped with restored PI3K signalling and the late phase of wound healing. Therefore, we next asked whether ERK and PI3K acted through Rho and Rac/Cdc42, respectively. At 10 minutes post injury, although control embryos showed clear activation of Rac (Fig. 6D), Cdc42 (Fig. 6E) and Rho (Fig. 6F), *spred1* overexpression (and thus ERK attenuation) caused hyper-activation of both Rac and Cdc42, whereas inhibition of PI3K signalling significantly reduced Rac and Cdc42 activation before and post wounding (Fig. 6D,E,G,H). When we examined Rho activation during wound healing, we found that *spred1* overexpression effectively reduced active Rho levels post wounding, whereas PI3K did not affect Rho (Fig. 6F,I).

To conclude, we identified Rho, Rac and Cdc42 as specific links between upstream ERK and PI3K signalling and downstream actin cytoskeletal dynamics. ERK signalling is responsible for Rho activation, which is known to regulate myosin-2 phosphorylation (Kimura et al., 1996). Meanwhile, PI3K activates Rac and Cdc42, which are known to be vital for the formation of lamellipodial and filopodial protrusions (Wood et al., 2002; Woolner et al., 2005). The higher levels of Rac and Cdc42 in ERK-inhibited embryos are probably induced by the restored PI3K activity, which also explains the increased filopodia formation seen in the early phase of wound closure when ERK is perturbed.

## Discussion

**Fig. 7.**
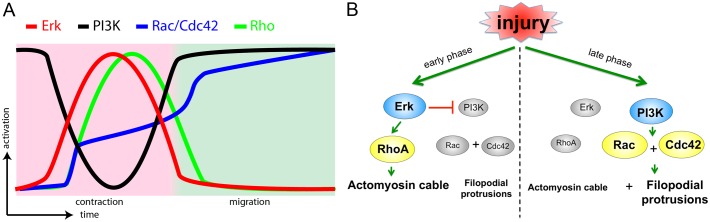
**Signal transduction in the fast and slow phases during wound healing.** (A) Schematic diagram showing the activation of ERK (red curve) and PI3K (black curve) signalling, small Rho GTPases Rac, Cdc42 (blue curve) and Rho (green curve) in contraction (pink background area) and migration (green background area) phases of embryonic wound healing. (B) Schematic diagram of early phase (left) and late phase (right) signalling of embryonic wound healing.

Target specificity and segregation in time and space are two major mechanisms that determine the efficiency and accuracy of signalling and their corresponding cellular behaviours. During embryonic wound healing, leading edge contraction and migration are two processes that share the same pool of cytoskeleton components and thus need to be exquisitely coordinated to ensure successful re-epithelialisation. In this study, we show that the fine-tuning of leading edge contraction and migration is achieved by grouping functionally different upstream signals into temporally distinct phases. In the first phase following injury, ERK signalling is activated, promoting downstream Rho activity and the phosphorylation of myosin-2 light chain, leading to the contraction of the actomyosin cable at the wound edge. During the second phase of closure, when migration and zippering take place, PI3K signalling activates its specific targets, Rac and Cdc42, thus facilitating actin accumulation and filopodia formation at the leading edge. In this way, we propose a new mechanism whereby target specificity and temporal segregation of signals are simultaneously incorporated in order to regulate distinct modes of cell motility in a complex setting, such as during embryonic wound healing (Fig. 7).

Previous studies in *Drosophila* wound closure also revealed distinct phases of wound healing, including a fast contraction phase promoted mainly by the actomyosin cable, and a slower zippering phase characterized primarily by extensive filopodial/lamellipodial protrusions (Wood et al., 2002; Abreu-Blanco et al., 2012). Intriguingly, tail-bud stage wound closure in *Xenopus* also shows a similar pattern of fast, then slow, dynamics (Fuchigami et al., 2011). Our findings demonstrate that the early *Xenopus* embryo, although containing relatively undifferentiated epidermal cells, still uses comparable mechanisms in wound healing to later stage *Xenopus* and *Drosophila* embryos, where the epithelia are more differentiated. However, a major difference between the *Xenopus* embryonic wound closure we describe here and *Drosophila* wound closure is that, whereas actin cable and protrusions assemble simultaneously at the wound edge in *Drosophila* (Wood et al., 2002; Abreu-Blanco et al., 2012), the two actin structures primarily form during different phases in *Xenopus* embryos. Interestingly, the distinct temporal separation of the different actin machineries has also been described during ventral enclosure in *C. elegans* embryos (Williams-Masson et al., 1997). Moreover, our observation that the contractile actin cable functions to restrict filopodial/lamellipodial migration and zippering closely resembles that seen during *Drosophila* dorsal closure (Jacinto et al., 2002). Given the similarities between cytoskeletal organization and regulation between our wound-healing system and these other morphogenetic processes, it is likely that the intracellular signalling mechanisms we have revealed during embryonic wound healing could be applicable more widely to other morphogenetic processes.

The formation of different actin structures is controlled by specific upstream regulators, such as the extensively studied small Rho GTPases Rac, Cdc42 and Rho. The temporally separate formation of actin structures that we observe in wound healing is associated with distinct activation profiles of these upstream small Rho GTPases. There are two possible mechanisms to accomplish this task. First, the small Rho GTPases could directly antagonise each other, as suggested during *Xenopus* oocyte wound healing, where Rho directly antagonises Cdc42 through the RhoGAP/GEF protein Abr (Vaughan et al., 2011). Second, the small Rho GTPases might not directly antagonise each other, but instead be separately regulated by temporally or spatially segregated upstream signals. Our results provide evidence for the second mechanism, where a temporal separation of upstream ERK and PI3K signalling determines the distinct activation profiles of Rho, Rac and Cdc42 following injury. Questions remain as to how this temporally sequential activation of ERK and PI3K signalling is achieved. It has been shown that growth factor stimulation can lead to similar sequential activation profiles of ERK and PI3K (Foschi et al., 1997). It is possible that a similar mechanism is involved in wound healing, even though it is still unclear what extracellular signals and plasma membrane receptors might activate Ras, ERK and PI3K in this context.

Most studies on the role of downstream targets of ERK signalling during wound healing focus on its activity at the transcriptional level, including driving the expression of growth factors, junctional proteins (Fitsialos et al., 2007) and key cytoskeletal regulators, such as Profilin (Brock et al., 2012). However, we find that phosphorylated ERK does not appear to exert its effect on embryonic wound healing through transcription, because inhibiting transcription does not affect the rate of healing.

When we turned to analyse the cytoplasmic role for pERK, we found evidence that it acts through Rho, leading to the phosphorylation of myosin-2. It has been shown that ERK MAPK phosphorylates a threonine residue on GEF-H1 (Fujishiro et al., 2008), a RhoGEF, under both growth factor and tension stimulations (Kakiashvili et al., 2009; Guilluy et al., 2011). Activation of GEF-H1 in turn enhances the activity of Rho and downstream phosphorylation of the myosin light chain (MLC) (Kakiashvili et al., 2009). In addition to this mechanism, it is also possible that ERK signalling phosphorylates another downstream target, myosin light chain kinase (MLCK), thus enhancing the activation of myosin-2 (Klemke et al., 1997). Because myosin-2 is the motor protein that drives actomyosin contraction, the major role of ERK activation that we observe in embryonic wound closure is to promote actomyosin contraction through activation of myosin-2. However, the unexpected finding that perturbing ERK signalling accelerates early phase wound closure and enhances wound edge protrusion and migration suggests a dual role of actomyosin contraction during wound healing, similar to the dual role of the purse string in *Drosophila* dorsal closure (Jacinto et al., 2002). It vigorously contracts the wound edge, but also restricts protrusive activity and migration at the same time. We suggest that besides contracting wound edges, the actomyosin cable might have another two benefits in the early phase, explaining why it is adopted as a major driving force even with the small price of delayed healing rate initially. First, it could enable a more organised tissue movement by reducing unnecessary cell mobilisation at the wound edge (Matsubayashi et al., 2011); and second, it might avoid possible premature activation of the contact-inhibition machinery that halts leading edge healing (Woolner et al., 2005). Moreover, in our study, we show that the actomyosin cable is required throughout two phases of wound closure, by pulling the wound edge taut and preventing a gaping-open wound.

We find that PI3K promotes Rac and Cdc42 activity during embryonic wound healing. Mechanistic links between PI3K and Rac/Cdc42 have been revealed as the PH-domain-containing GEFs, such as Sos (Nimnual et al., 1998; Soisson et al., 1998), Tiam-1 (Fleming et al., 2000), SWAP-70 (Shinohara et al., 2002), aPIX (Yoshii et al., 1999), DOCK180 (Côté and Vuori, 2002), Vav (Han et al., 1998) and Zizimin1 (Meller et al., 2002). It will be very interesting to investigate which of the GEFs mediates signalling from PI3K to Rac and Cdc42 during embryonic wound healing. Furthermore, we find that the level of PI3K signalling is largely correlated to the amount of filopodia: hyperactivation of PI3K induces increased protrusions, wild-type PI3K activity shows intermediate filopodia number, and inactivation causes a protrusive defect. We also find that PI3K signalling is required for F-actin accumulation at the wound margin. Because the preferred formation of dynamic actin structures at the leading edge suggests polarisation of wound-edge cells, the effect of PI3K on both filopodia formation and actin accumulation implies another role of this pathway. It could be that PI3K functions to establish an intracellular gradient of its product, phosphatidylinositol 3,4,5-trisphosphate (PIP3), which determines the polarity of leading edge cells and the directionality of cell migration (Funamoto et al., 2002; Pickering et al., 2013).

Our findings also have two more implications. First, we find that in different phases of embryonic wound healing, the purse string mechanism and actin-protrusion-guided migration and zippering both exist. The former is considered as a major driving force in embryonic wound closure, whereas the latter dominates the majority of adult wounds. We find that by modulating ERK and PI3K signal transduction pathways, we are able to alter the mode of tissue movement in embryonic wound healing. For example, high ERK and low PI3K induces the actomyosin contraction mode, and high PI3K and low ERK shifts the contraction mode to a more-protrusive migration mode. Given that embryos coordinate these signalling pathways and cellular behaviours such that perfect, scarless wound healing is achieved, it is intriguing to speculate whether mimicking the same dynamics in adults facilitates a better healing outcome in adult patients suffering from acute or chronic wounds.

## Materials and Methods

### Embryo culture and manipulation

Embryo culture was performed as described (Sive et al., 2000). Capped mRNA was injected at the 1- or 2-cell stage to all cells in a total volume of 10 nl. Amounts injected were 500 pg *spred1* mRNA (Sivak et al., 2005), 500 pg (medium dose) or 1 ng (high dose) DN PI3K (*Δp85*) mRNA (Carballada et al., 2001), 50 pg CA PI3K (*p110 CAAX*) mRNA (Carballada et al., 2001), 200 pg *gfp*/*mcherry*-*moesin* mRNA, 500 pg *gfp*-*grp1* (PH domain) mRNA, 500 pg *GST-PAK1* mRNA (70-110) (Addgene, Cambridge, MA), 500 pg *GST-rGBD* mRNA (Clark et al., 2009) and 150 ng *Xenopus* RhoA mRNA [EST library (Gilchrist et al., 2004)]. Inhibitors PD184352 (Sigma-Aldrich) and LY294002 (Cell Signaling Technology) were made into stocks in DMSO, and diluted in 75% (v/v) normal amphibian medium (NAM) [110 mM NaCl, 2 mM KCl, 1 mM Ca(NO_3_)_2_, 1 mM MgSO_4_, 0.1 mM EDTA, 1 mM NaHCO_3_, 2 mM sodium phosphate, pH 7.4] to 10 µM PD and 25 or 50 µM LY before treating embryos. 0.1% DMSO was used as control for the drug treatments.

### Immunofluorescence and hematoxylin staining

Embryos were fixed with MEMFA (Sive et al., 2000), embedded in 16.7% fish gelatin (20 ml 40% gelatin (Sigma-Aldrich), 7.5 g sucrose in 50 ml ddH_2_O) and sectioned with Cryostat Leica CM3050. Samples were permeabilised with 100% acetone and re-hydrated with TBS. For immunofluorescence, sections were blocked in TBST with BSA or milk, before antibody incubation. Primary antibodies were used at 1∶500 pERK (Cell Signaling Technology) in TBST with BSA, 1∶200 β-catenin (Abcam) in TBST with milk and 1∶200 Ser19 Myosin II Light Chain (Cell Signaling Technology) in TBSN with BSA, 1∶10,000 DAPI in TBST with BSA at 4°C overnight. Samples were then stained with Alexa Fluor 488 or 568 secondary antibodies (Molecular Probes) at 1∶500 in TBSN with BSA for 1 hour at room temperature. Samples were mounted with AntiFade Gold (Invitrogen) for imaging. For hematoxylin staining, slides were stained with 50% hematoxylin solution (Sigma-Aldrich), washed with tap water, and differentiated with 0.1% HCl. The ethanol-Histoclear method was applied to dehydrate samples before mounting with DPX reagent (Fisher).

### Wounding and imaging

Late blastula stage wild-type *Xenopus laevis* embryos (stage 9–9.5) were superficially (for whole embryo wound closure imaging and F-actin imaging) or deeply (for western blot, pull-down and immunofluorescence) wounded on the animal hemisphere with forceps, in ¾ NAM solution at room temperature (Davidson et al., 2002). Time-lapse images were captured with the 80× objective on a Leica M205 microscope with Leica AF software (Leica Microsystems, Heerbrugg, Switzerland). Images were processed with NIH ImageJ. Wound closure percentage  =  (1–wound area at a certain time point)/starting wound area. For imaging F-actin dynamics or PIP3 localisation, albino *Xenopus laevis* embryos were injected with *gfp*-*moesin* mRNA or *gfp*-*grp1* mRNA, respectively. At blastula stage, the whole animal caps were extracted in ¾ NAM solution, wounded by forceps on the superficial layer, and imaged with a 20× objective Olympus IX81 confocal microscope with Fluoview software. Images were processed with NIH ImageJ.

### GST pull down

Active Rac, Cdc42 and Rho pull down was performed as described previously (Soto et al., 2013).

### Western blot

Ten embryos were collected for each sample, cooled in dry ice and stored at −80°C until extraction. Frozen embryos were homogenized using freshly prepared PK buffer, 50 µl buffer for 10 embryos (Dorey and Hill, 2006). Homogenized lysates were centrifuged at 4°C, 13,200 rpm for 15 minutes to separate clear lysate from precipitated yolk. Clear lysate was aspirated and immediately mixed well with equal volume of 2× SDS loading buffer, boiled at 98°C for 2.5 minutes and frozen at −80°C until use. 10 µl protein extract was loaded on each well of the 8% SDS-PAGE gel, and transferred onto PVDF membranes. The membranes were blocked with 5% BSA in TBST overnight at 4°C. Primary antibodies were used at a dilution of 1∶10,000 pERK (Cell Signalling Technology), 1∶1000 pAkt (Cell Signaling Technology) and 1∶100,000 α-tubulin (Sigma-Aldrich). For GST pull down, primary antibodies were used at 1∶2000 Rac1 (Cell Signalling Technology), 1∶1000 Cdc42 (Cell Signalling Technology) and 1∶1000 RhoA (Santa Cruz Biotechnology, Santa Cruz, CA) in TBST with BSA. Secondary antibodies were used at a dilution of 1∶40,000 anti-Rabbit (Dako) or 1∶100,000 anti-Mouse (Dako, Glostrup, Denmark). Signal detection was carried out using the Immobilon™ Western HRP substrate on X-ray films (Milipore, Billerica, MA).

### Quantification and statistics

To quantify F-actin and phosphorylated myosin-2 accumulation at the wound edge, we measured F-actin (GFP-moesin) or pMLC fluorescence intensity (FI) across a cell at the wound edge. Relative FI shows the ratio of F-actin or pMLC FI at the leading edge plasma membrane relative to the F-actin or pMLC FI on the distal side plasma membrane (leading edge FI/distal side FI). For protein gel quantification, X-ray films from three independent experiments were scanned into high-resolution images, and the density of each protein band was determined by ImageJ Gel Quantification Tool. To compensate for the variation of protein band density between different experiments, the percentage value of each band of the total protein density from the same gel calculated by the ImageJ Gel Quantification Tool was used, instead of the absolute density of each protein band. The quantities of pERK and pAkt bands were normalised with α-tubulin controls. The quantities of active GTPases and respective inputs were normalised with GST loading controls, and the relative intensity of each active GTPase pull-down band is the ratio of pull-down GTPase to input GTPase, which indicates the percentage of active GTPase in total GTPase. Statistical analysis used for each experiment was specified in related figure legends.

### Constructs

The binding domain of moesin (Millard and Martin, 2008) and the PH domain of Grp1 (Pickering et al., 2013) were gifts from Tom Millard and subcloned into pDEST and pCS107 vectors with eGFP/mCherry at the N-terminus, respectively. The dominant-negative PI3K (Δp85) and constitutively active PI3K (p110 CAAX) were gifts from Laurent Kodjabachian (Carballada et al., 2001). Full-length *Xenopus* Rac1, Cdc42 and RhoA cDNAs were obtained from the Gurdon Institute *Xenopus* tropicalis EST library (Gilchrist et al., 2004) (http://genomics.nimr.mrc.ac.uk/online/xt-fl-db.html), as constructs in vector pCS107 (Rac1, TEgg073116; Cdc42, TEgg040f06; RhoA, TEgg071k05) and subcloned into pCS107 vector. For GST pull-down assay, pGEXTK-PAK1 70–117 (GST-PAK) plasmid was purchased from Addgene (Addgene plasmid 12217, provided by Jonathan Chernoff, Fox Chase Cancer Center, PA). RBD of Rhotekin (rGBD) was a kind gift from B. Bement (Benink and Bement, 2005). Both probes were subcloned into pCS107. GST was subcloned from pGEXTK-PAK1 plasmid into pCS107, at the C termini of PAK1 and rGBD.

Primers were used as follows: Full-length *Xenopus* Rac1, forward: ATCGGAATCGAT ATGCAGGCCATTAAATGTGTGGT and reverse: ATCCGTCGAC TTATTACAACAGCAGGCATTTTCT; Full-length *Xenopus* Cdc42, forward: ATCGGAATCGAT ATGCAGACAATTAAATGTGTAGT and reverse: ATCCGTCGAC TCATAGCAGCCTACACTTGCGTTT; Full-length *Xenopus* RhoA, forward: ATCGGAATCGAT ATGGCAGCCATCCGTAAGAAGCTT and reverse: ATCCGTCGAC TTAGATGAGAAGGCACGTGGTTTT; pak1 70-117, forward: ATCGGAGAATTC AAAGAGCGGCCAGAGATTTCTCT and reverse: ATCCGCTAGC CTGCTCCGACTTAGTGATATTT; rGBD, forward: ATCGGAGAATTCatcctggaggacctcaatatgctcta and reverse: ATCCGCTAGCgcctgtcttctccagcacctgggcct; GST, forward: ATCGGAATCGAT atgTCCCCTATACTAGGTTATTGGaa and reverse: ATCCGTCGAC ctaTCCACGCGGAACCAGATCCGA.

## Supplementary Material

Supplementary Material
