# Supplementary material for: ERK and phosphoinositide 3-kinase temporally coordinate different modes of actin-based motility during embryonic wound healing
Source: J Cell Sci. 2013 Nov 1;126(21):5005–17. doi: 10.1242/jcs.133421 (PMC3820245; doi:10.1242/jcs.133421)
Supplement: Supplementary Material [file supp_126_21_5005__index.html]

ERK and phosphoinositide 3-kinase temporally coordinate different modes of actin-based motility during embryonic wound healing — Supplementary Material 

# ERK and phosphoinositide 3-kinase temporally coordinate different modes of actin-based motility during embryonic wound healing

## JCS133421 Supplementary Material

**Files in this Data Supplement:**

- **Supplementary Material PDF**
