# Supplementary material for: ERK and phosphoinositide 3-kinase temporally coordinate different modes of actin-based motility during embryonic wound healing
Source: J Cell Sci. 2013 Nov 1;126(21):5005–17. doi: 10.1242/jcs.133421 (PMC3820245; doi:10.1242/jcs.133421)
Supplement: Supplementary Material [file supp_126.21.5005_JCS133421.pdf]

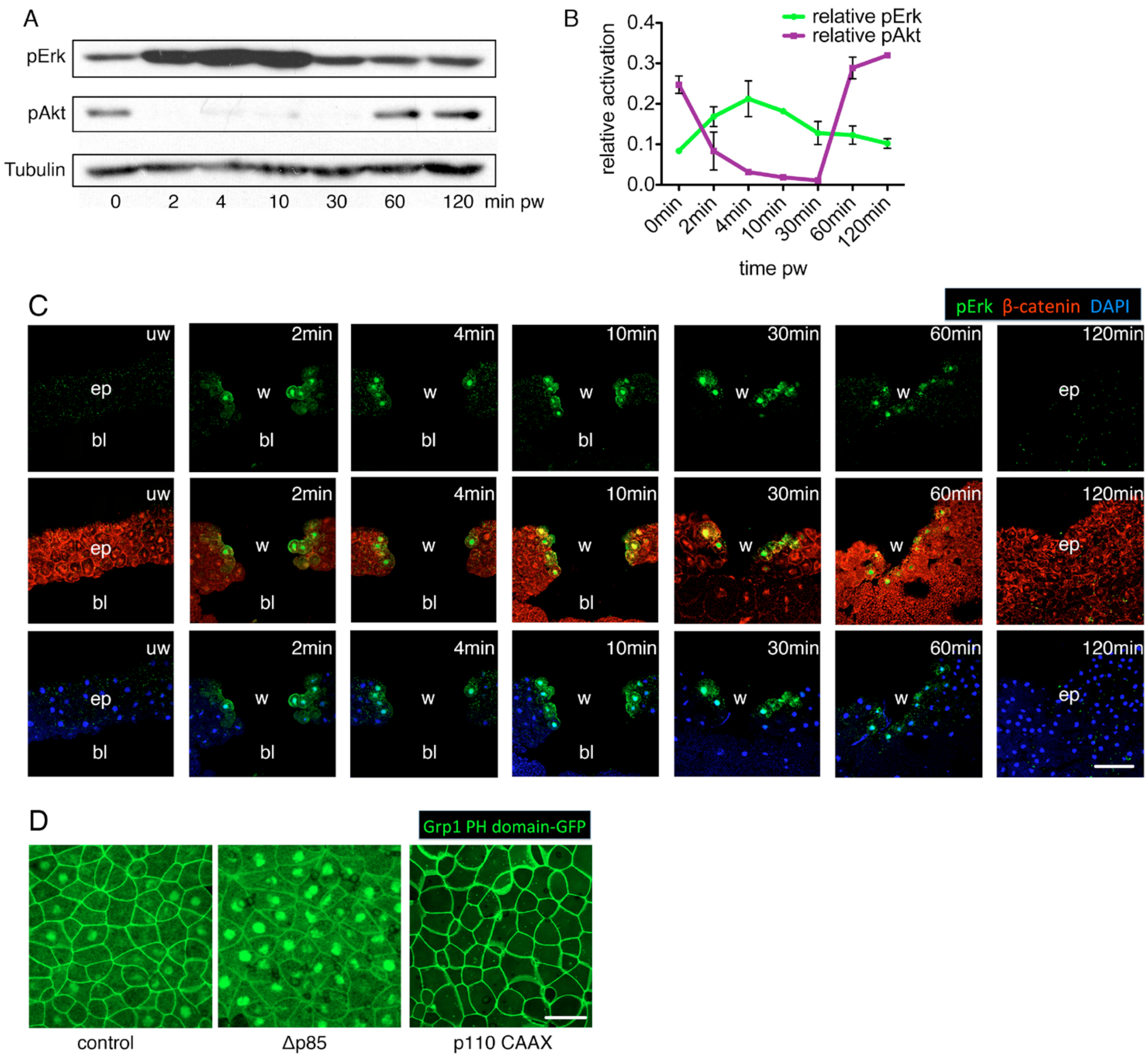

Fig. S1. Erk and PI3K signalling during embryonic wound healing (Related to Fig. 2). (A) Western blot analysis shows the sequential activation of pErk and pAkt in superficial wounds, with α-tubulin as loading control. (B) Quantification of pErk and pAkt activation during embryonic wound healing. Data were collected from two independent experiments. pErk and pAkt signal intensities were normalized to α-tubulin controls. (C) Immunofluorescence staining of pErk, β-catenin (plasma membrane) and DAPI (nucleus) on transected embryonic wounds, complete time course post wounding. w: wound. bl: blastocoel. ep: epithelium. Scale bar represents 50 μm. (D) Control experiments using the GFP-Grp1 probe, showing that PIP3 accumulates at the plasma membrane with a moderate intensity in control embryos relative to Δ*p85* injected embryo, which display a weak PIP3 signal on the membrane and *p110 caax* injected embryo, which shows a strong and membrane-specific accumulation of PIP3. Scale bar: 20 μm.

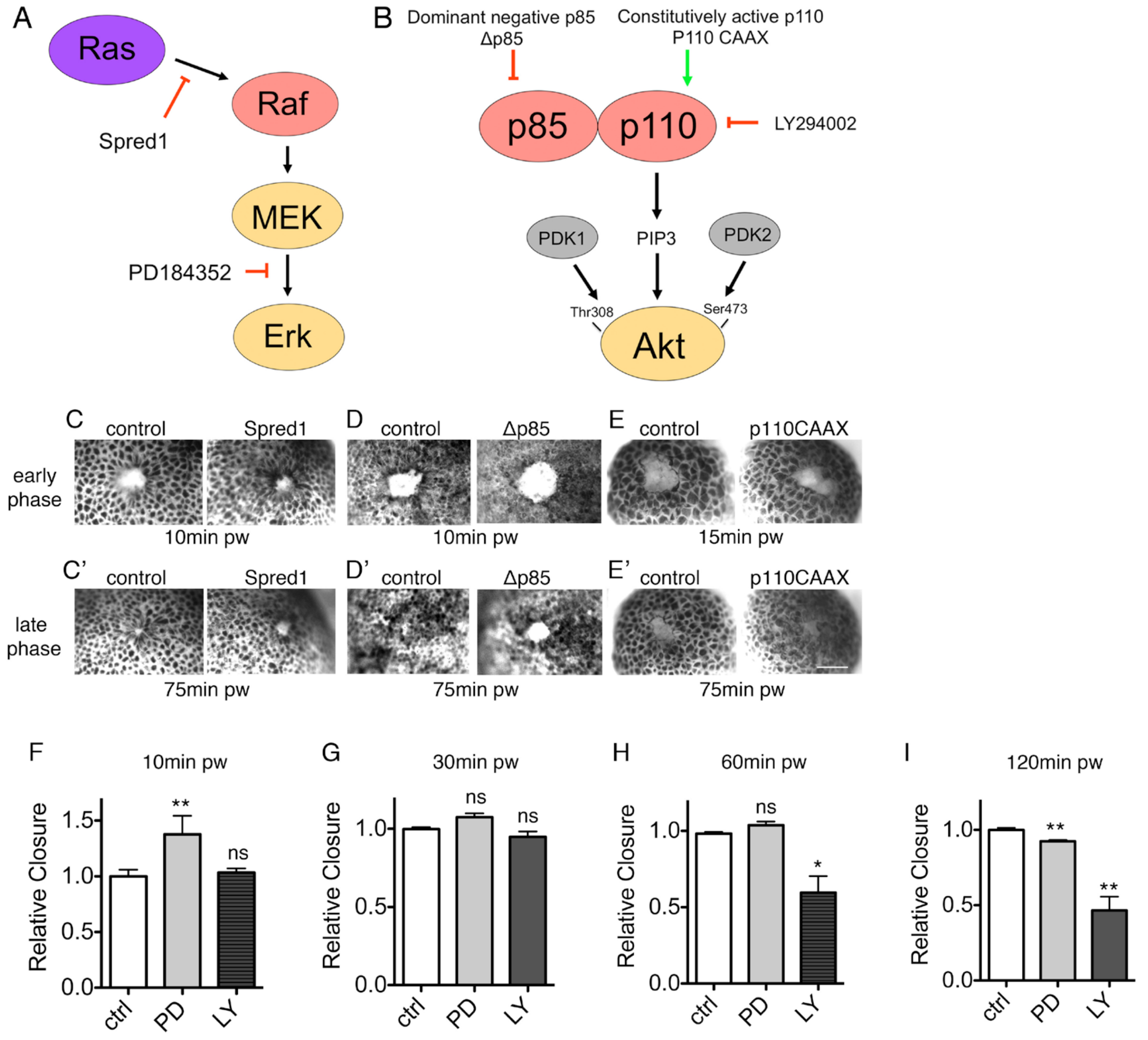

Fig. S2. Erk and PI3K signalling regulate distinct phases of wound healing (Related to Fig. 3). (A) Schematic diagram of Ras-Erk pathway. Spred1 disrupts signal transduced from Ras to Raf, PD184352 inhibits MEK activity. (B) Schematic diagram of PI3K signalling. Δp85 is the dominant negative form of the regulatory subunit of PI3K, p110 CAAX is the constitutively active form of the catalytic subunit of PI3K, LY294002 specifically inhibits the catalytic subunit of PI3K. Akt phosphorylation requires PIP_3 guided translocation of PI3K to the plasma membrane, PDK1 and PDK2 phosphorylates Thr308 and Ser473, respectively. (C-E) Magnified view of *spred1* (C-C'), *Δp85* (D-D') and *p110 caax* (E-E') injected embryos at key time points post wounding in parallel comparison with controls. Scale bar: 200 μm. (F-I) Quantifications of relative wound closure of PD and LY treated embryos 10 min (C), 30 min (D), 60 min (E) and 120 min (F) post wounding. Mean wound closure percentage of control embryos was normalized to 1; other groups show relative closure against control. Non-parametric Mann Whitney test was used to test for significance between control and other groups. *p<0.05. **p<0.01. ***p<0.001. ns: not significant. Results are shown as means ± SEM from at least three independent experiments. For each experiment, at least 3 embryos were taken for each group.

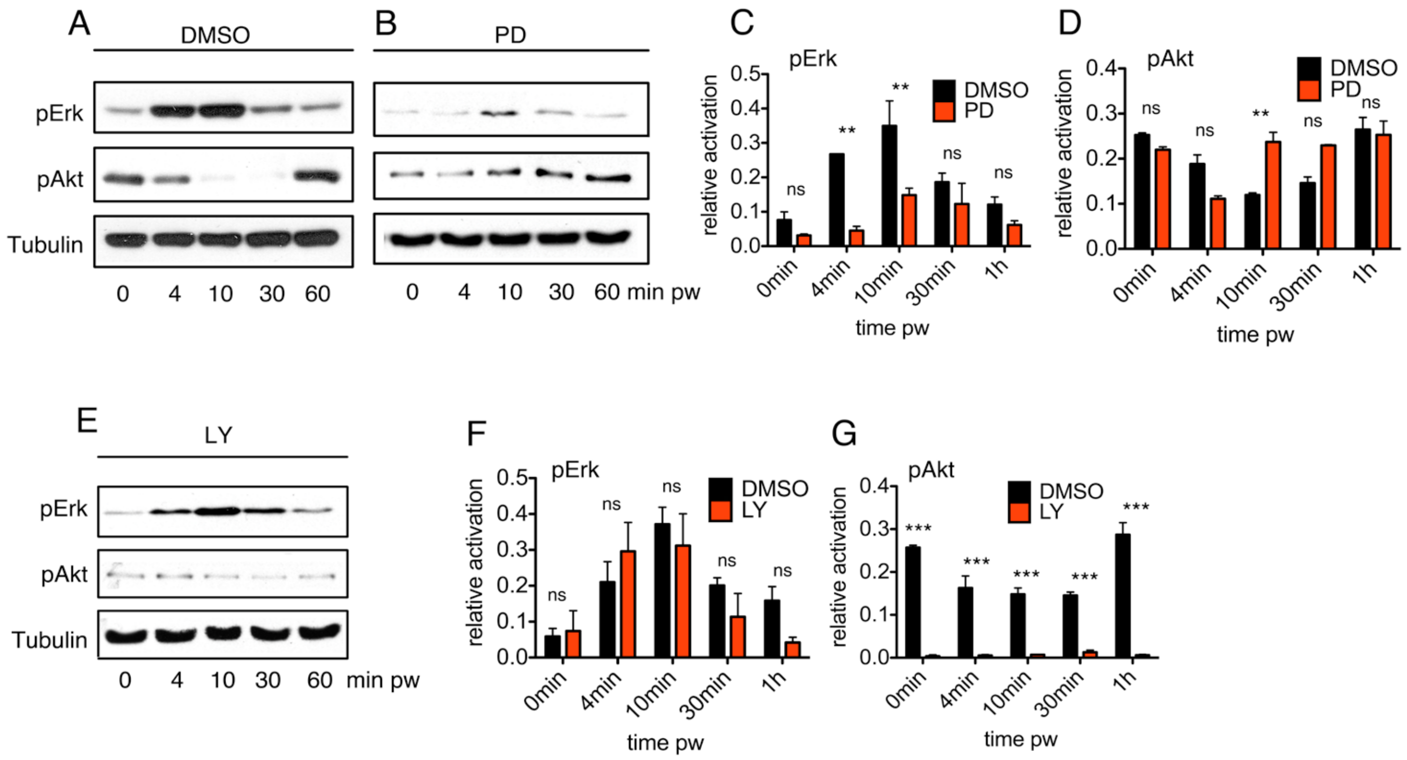

Fig. S3. Erk inhibition results in a quicker restoration of PI3K signalling (Related to Fig. 4). (A-D) Western blot and quantification of pErk and pAkt in DMSO (A) and PD184352 (PD) (B) treated embryos during embryonic wound healing, showing that PD significantly reduces Erk activation post wounding (C), but pAkt is restored quicker, 10 min pw (D). (E-G) Western blot and quantification of pErk and pAkt in LY294002 (LY) treated embryos during embryonic wound healing, showing that LY significantly reduces pAkt (G) level, but pErk (F) level is not affected. Quantification was performed on data collected from three independent experiments, signal intensities were normalized to α-tubulin controls and analysed by ImageJ. pw: post wounding. *p<0.05. **p<0.01. ***p<0.001. ns: not significant.

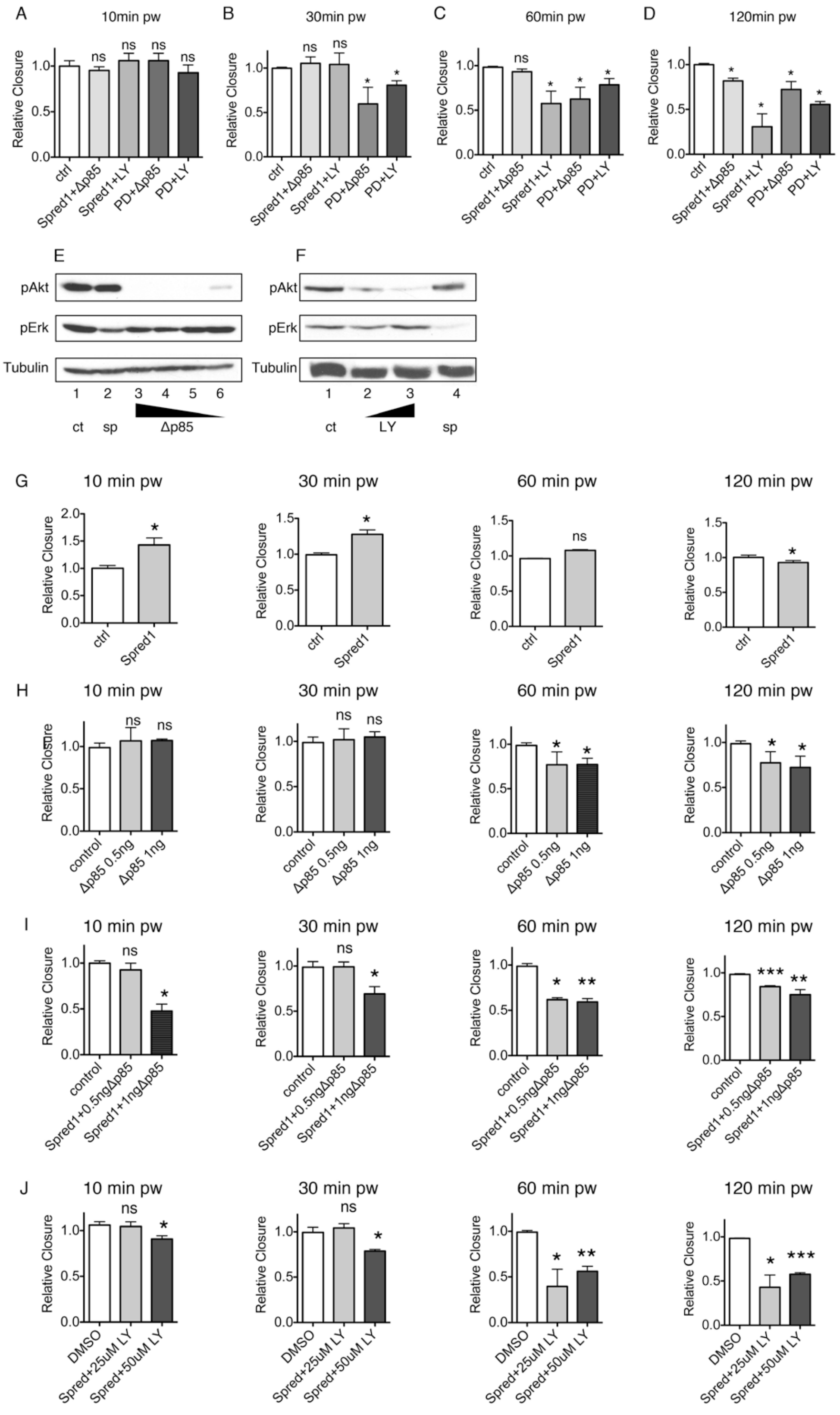

Fig. S4. PI3K drives embryonic wound closure in the absence of Erk signalling (Related to Fig. 4). (A-D) Quantification of wound closure in control and combined inhibition of Erk and PI3K signalling. Both Δp85 and LY were used at a medium concentration. (E-F) Western blot showing PI3K inhibition with a gradient dose of Δp85 and LY. ct: control. sp:Spred1. (E) Lane 3: 2ng Δp85. Lane 4: 1.5ng Δp85. Lane 5: 1ng Δp85. Lane 6: 0.5ng Δp85. (F) Lane 2: 25μM LY. Lane 3: 50μM LY. (G-J) Quantification of relative wound closure speed in Spred1 (G), different doses of Δp85 (H), Spred1 combined with different doses of Δp85 (I), Spred1 combined with different doses of LY (J). Mean wound closure in control embryos was normalized to 1; other groups show relative closure against control. Non-parametric Mann Whitney test was used to test for significance between control and other groups. *$p<0.05$. **$p<0.01$. ***$p<0.001$. ns: not significant. Results are shown as means ± SEM from at least three independent experiments. For each experiment, at least 3 embryos were taken for each group.

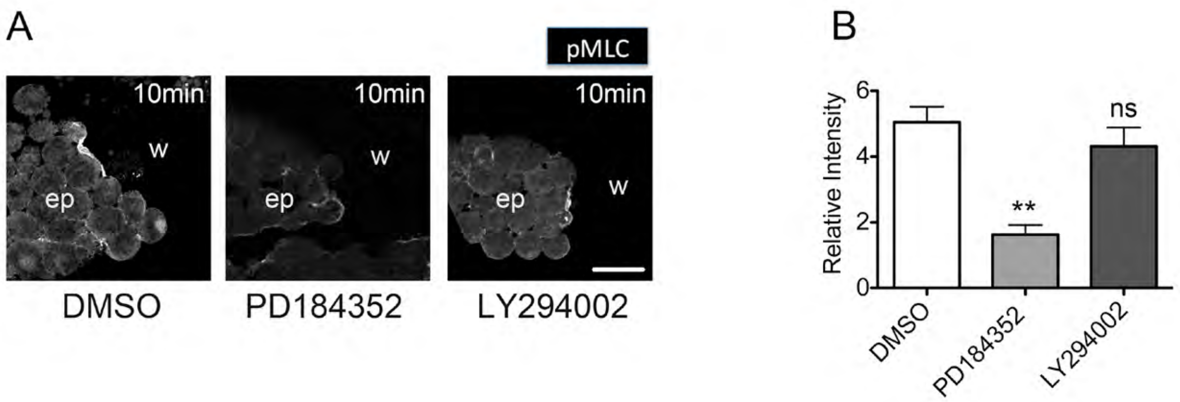

Fig. S5. Erk signalling is responsible for myosin-2 phosphorylation at the wound edge (Related to Fig. 5). (A) pMLC immunofluorescence staining in DMSO, PD and LY treated embryos (transected across the wound edge) 10 min post wounding. w: wound. Scale bar represents 50 µm. (B) Quantification of relative pMLC fluorescence intensity at the wound edge. n = 20 cells were measured. Results are shown as means ± SEM. Kruskal-Wallis one way ANOVA test was used compare all groups to control. **p<0.01. ns: not significant.

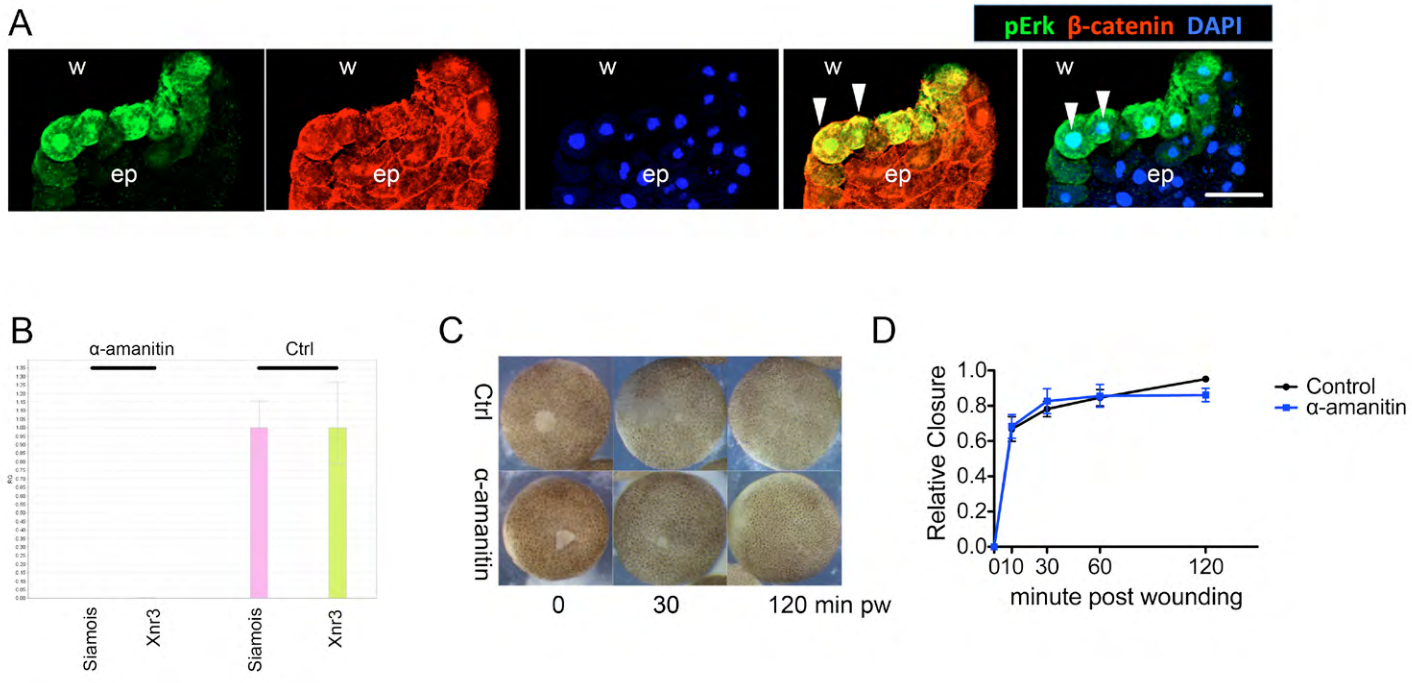

Fig. S6. Erk and PI3K signaling do not regulate wound healing on the transcriptional level (Related to Fig. 6). (A) Confocal image of immunofluorescence staining of pErk (green), β-catenin (red) and DAPI (blue) on transected embryonic wound, showing colocalisation of pErk and β -catenin (plasma membrane), pErk and DAPI (nucleus) in wound edge cells. Embryo was fixed 10 min post wounding for sections. Arrows show colocalisations. Scale bar represents 50 µm. (B) Real-time PCR of *xnr3* and *siamois* at gastrula stage in control and α-amanitin injected embryos, indicating the effectiveness of α-amanitin in abolishing transcription. (C) Images of embryonic wound closure in control and α-amanitin injected embryos. pw: post wounding. (D) Quantification of wound closure in control and α-amanitin injected embryos. Data were representative of three independent experiments. Two-way ANOVA was performed to confirm significance.

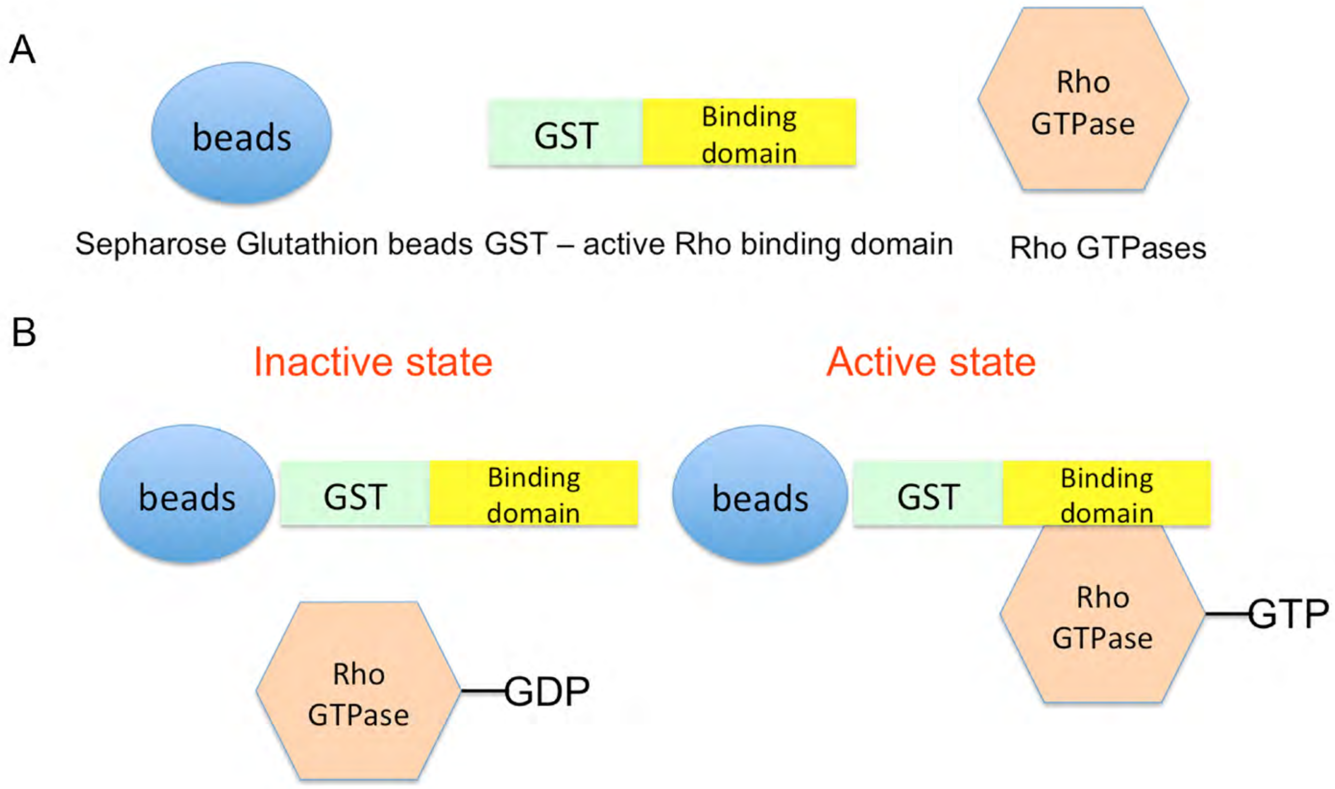

Fig. S7. Active Rho GTPase pull down assay (Related to Fig. 6). (A) Schematic diagram of critical components in active Rho GTPase GST pull down. (B) Principle of active Rho GTPase GST pull down.

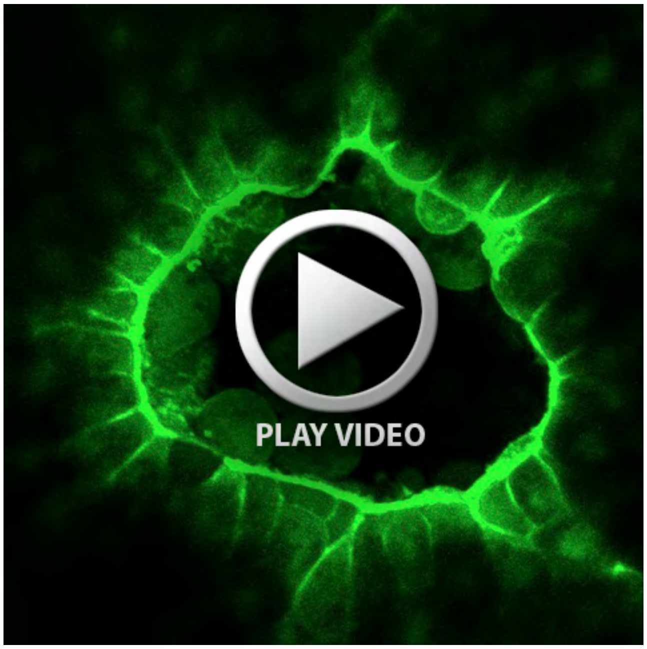

Movie 1. Time-lapse movie of actin cable formation in control wounded embryo (Related to Fig. 1). Movie shows formation of circumferential actin ring at the wound edge. Movie starts from 10 min post wounding. Embryo was injected with mRNA encoding GFP-moesin (actin binding domain) and wounded at St.10. Images were taken every 30 sec. 10 frames.

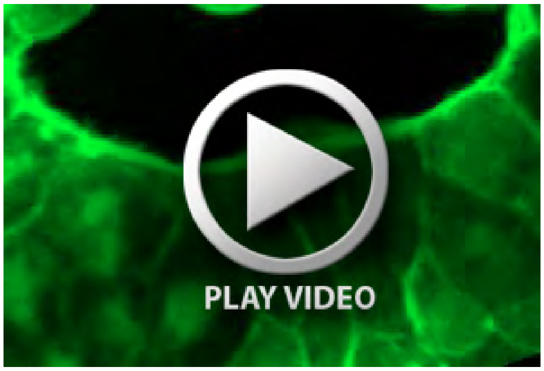

Movie 2. Time-lapse movie of actin cable formation in control wounded embryo (Related to Fig. 1). Movie shows formation of a half actin ring at the wound edge. Movie starts from 10 min post wounding. Embryo was injected with mRNA encoding GFP-moesin (actin binding domain) and wounded at St.10. Images were taken every 30 sec. 15 frames.

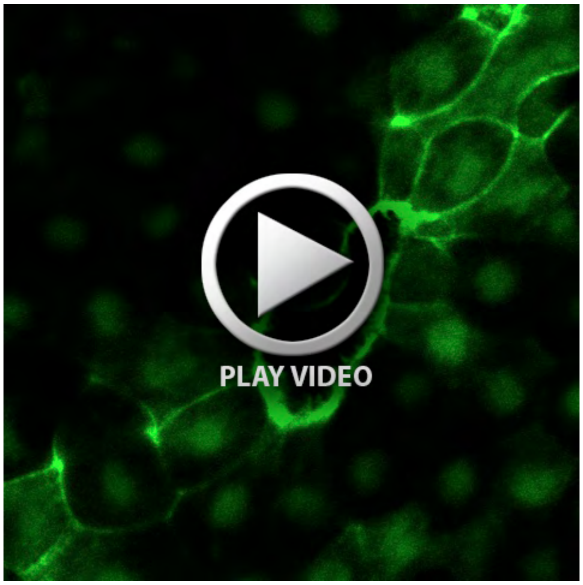

Movie 3. Time-lapse movie of control embryo showing filopodia formation at the proximal wound edge (Related to Fig. 1). Movie starts from 60 min post wounding. Embryo was injected with mRNA encoding GFP-moesin (actin binding domain) and wounded at St.10. Images were taken every 30 sec. 20 frames.

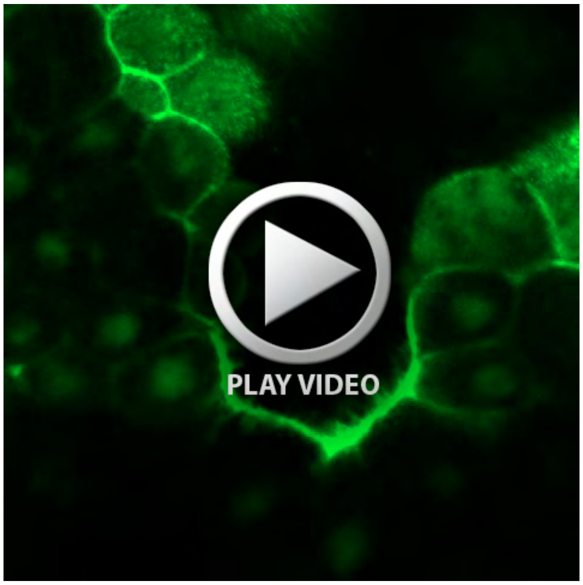

Movie 4. Time-lapse movie of control embryo showing long filopodia formation at the proximal wound edge (Related to Fig. 1). Movie starts from 60 min post wounding. Embryo was injected with mRNA encoding GFP-moesin (actin binding domain) and wounded at St.10. Images were taken every 30 sec. 20 frames.

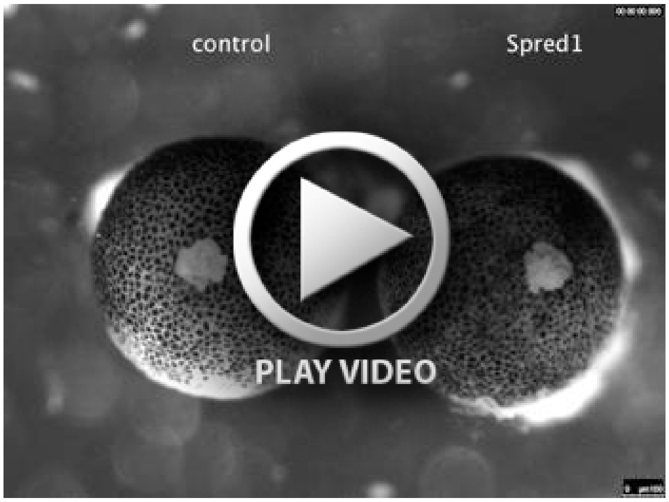

Movie 5. Time-lapse movie of control and *spred1* mRNA injected embryos during wound healing (Related to Fig. 3). Wounds were made on St.9 embryos. Left: control embryo. Right: Spred1 injected embryo. Pictures were taken per 20 sec for a total of 98 min.

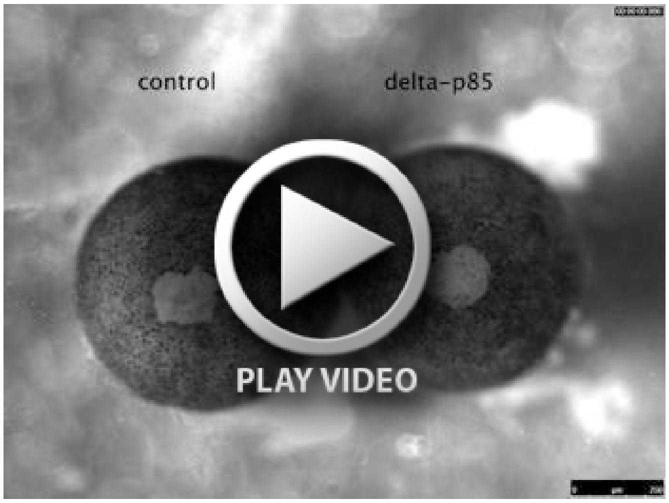

Movie 6. Time-lapse movie of control and Δ*p85* mRNA injected embryos during wound healing (Related to Fig. 3). Wounds were made on St.9 embryos. Left: control embryo. Right: Δp85 injected embryo. Pictures were taken per 30 sec for a total of 87 min.

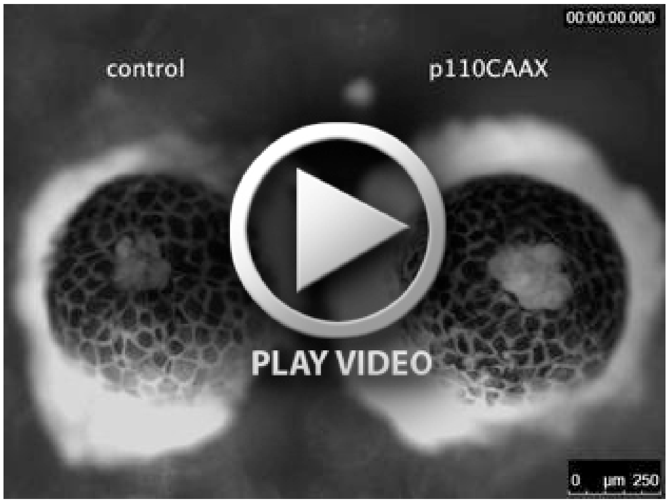

Movie 7. Time-lapse movie of control and *p110 caax* mRNA injected embryos during wound healing (Related to Fig. 3). Wounds were made on St.8 embryos. Left: control embryo. Right: p110 caax injected embryo. Pictures were taken per 30 sec for a total of 180 min.

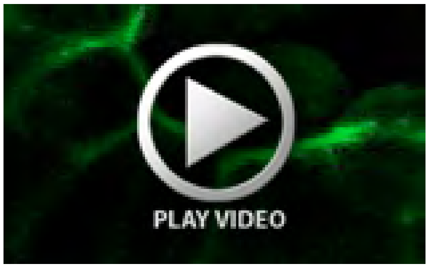

Movie 8. Time-lapse movie of actin cable formation at the edge of a control wounded embryo (Related to Fig. 5). Note that as the actin cable forms, only a few filopodia extend from the leading edge. Movie starts from 9.5 min post wounding. Embryo was injected with mRNA encoding GFP-moesin (actin binding domain) and wounded at St.10. Images were taken per 15 sec. 10 frames.

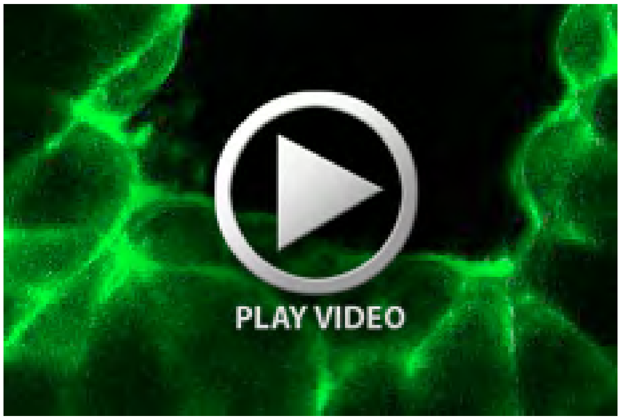

Movie 9. Time-lapse movie of actin cable formation at the edge of *spred1* mRNA injected wounded embryo, showing a dramatic increase in filopodia formation at the leading edge (Related to Fig. 5). Movie starts from 10 min post wounding. Embryo was injected with mRNA encoding GFP-moesin (actin binding domain) and wounded at St.10. Images were taken per 15 sec. 10 frames.

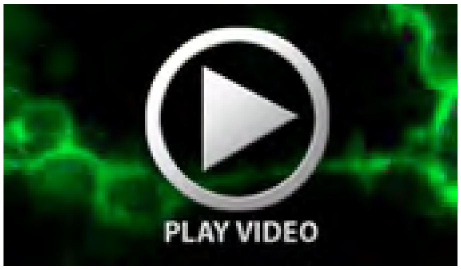

Movie 10. Time-lapse movie of actin cable formation at the edge of *p110 caax* mRNA injected wounded embryo, showing s dramatic increase in filopodia formation at the leading edge (Related to Fig. 5). Movie starts from 8 min post wounding. Embryo was injected with mRNA encoding GFP-moesin (actin binding domain) and wounded at St.10. Images were taken per 15 sec. 10 frames.

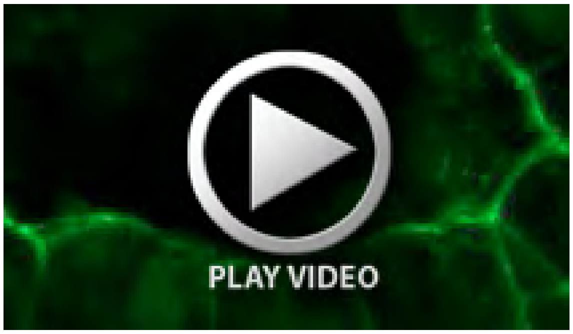

Movie 11. Time-lapse movie of actin cable formation at the edge of Δ*p85* mRNA injected wounded embryo, showing impaired actin cable formation and filopodia formation (Related to Fig. 5). Movie starts from 9 min post wounding. Embryo was injected with mRNA encoding GFP-moesin (actin binding domain) and wounded at St.10. Images were taken per 15 sec. 10 frames.
